# miRNAs Potentially Involved in Post Lung Transplant-Obliterative Bronchiolitis: The Role of miR-21-5p

**DOI:** 10.3390/cells10030688

**Published:** 2021-03-20

**Authors:** Sara Bozzini, Laura Pandolfi, Elena Rossi, Simona Inghilleri, Michele Zorzetto, Giuseppina Ferrario, Stefano Di Carlo, Gianfranco Politano, Annalisa De Silvestri, Vanessa Frangipane, Michele Porzio, Romain Kessler, Fiorella Calabrese, Federica Meloni, Patrizia Morbini

**Affiliations:** 1 Research Laboratory of Lung Diseases, Section of Cell Biology, IRCCS Policlinico San Matteo Foundation, 27100 Pavia, Italy; s.bozzini@smatteo.pv.it (S.B.); l.pandolfi@smatteo.pv.it (L.P.); simona20ago@gmail.com (S.I.); m.zorzetto@smatteo.pv.it (M.Z.); v.frangipane@smatteo.pv.it (V.F.); f.meloni@smatteo.pv.it (F.M.); 2 Department of Molecular Medicine, University of Pavia, 27100 Pavia, Italy; elena.rossi@unipv.it (E.R.); giuseppina.ferrario01@universitadipavia.it (G.F.); 3 Medical Genetics Unit, IRCCS Mondino Foundation, 27100 Pavia, Italy; 4 Control and Computer Engineer Department, Politecnico di Torino, 20129 Torino, Italy; stefano.dicarlo@polito.it (S.D.C.); gianfranco.politano@polito.it (G.P.); 5 Clinical Epidemiology and Biometrics Unit, Foundation IRCCS Policlinico S. Matteo, 27100 Pavia, Italy; a.desilvestri@smatteo.pv.it; 6 Service de Pneumologie, NouvelHôpital Civil, Hôpitaux Universitaires de Strasbourg, Fédération de Médecine Translationnelle (FMTS), 67085 Strasbourg, France; michele.porzio@chru-strasbourg.fr (M.P.); Romain.Kessler@chru-strasbourg.fr (R.K.); 7 Department of Cardiovascular and Thoracic Sciences, University of Padova, 35122 Padova, Italy; fiorella.calabrese@unipd.it; 8 Department of Internal Medicine, University of Pavia, 27100 Pavia, Italy

**Keywords:** chronic lung allograft dysfunction, bronchiolitis obliterans, microRNA, miR-21-5p, in situ hybridization

## Abstract

Epigenetic changes, including miRNAs deregulation, have been suggested to play a significant role in development of obliterative bronchiolitis (OB) in transplanted lungs. Many studies have tried to identify ideal candidate miRNAs and the downstream pathways implicated in the bronchiolar fibro-obliterative process. Several candidate miRNAs, previously indicated as possibly being associated with OB, were analyzed by combining the quantitative real time-polymerase chain reaction (qRT-PCR) and in situ hybridization (ISH) of lung tissues of OB affected patients. Disease and OB-lesion-specific expression of miR-21-5p was confirmed and by computational analysis we were able to identify the network of genes most probably associated miR-21-5p in the context of OB fibrogenesis. Among all potentially associated genes, STAT3 had a very high probability score. Immunohistochemistry showed that STAT3/miR-21-5p were co-over expressed in OB lesions, thus, suggesting miR-21-5p could regulate STAT3 expression. However, miR-21-5p inhibition in cultures of bronchiolitis obliterans syndrome (BOS) derived myofibroblasts did not significantly affect STAT3 mRNA and protein expression levels. This study demonstrates the specificity of miR-21-5p over-expression in OB lesions and contributes to existing knowledge on the miR-21-5p downstream pathway. Activation of STAT3 is associated with miR-21-5p upregulation, however, STAT-3 network activation is most likely complex and miR-21-5p is not the sole regulator of STAT3.

## 1. Introduction

Lung transplantation (LTx) is an effective therapy for many end-stage lung diseases. Chronic lung allograft dysfunction (CLAD), however, continues to limit long-term survival and presents with two, frequently overlapping, major clinical phenotypes: bronchiolitis obliterans syndrome (BOS) and restrictive allograft syndrome (RAS) [1]. BOS accounts for roughly 70–80% of cases and represents the main cause of long-term morbidity and mortality. BOS is the result of an inflammatory and fibrotic process inducing the occlusion of small airways, but its pathogenesis has not been clarified yet [2]. The histologic correlate of BOS is obliterative bronchiolitis (OB), which consists of bronchiolar segmental submucosal fibrosis that causes total and irreversible occlusion of the airway lumina [3]. RAS corresponds to a pattern of interstitial fibrosis mostly resembling non-specific interstitial pneumonia (NSIP) and/or pleuropulmonary fibroelastosis at histopathological examination. There is no effective treatment for CLAD and pharmacological therapy is often unsuccessful once functional impairment has developed. Studies aiming to clarify the mechanisms underlying OB development have suggested that, in addition to alloimmune and inflammatory phenomena centered in the small airways, microRNA (miRNA) dysregulation, aberrant DNA methylation and histone post-translational modifications may be relevant in BOS pathogenesis [4]. miRNAs are short, non-coding RNAs composed of 15–22 nucleotides that act as translational repressors and intervene in the control of gene expression. Since their discovery, alterations of miRNA have been described in almost all human diseases, including neoplastic, metabolic, inflammatory, and neurological disorders, as well as in cardiovascular and autoimmune diseases [5,6]. In particular, miRNAs have been found to play a significant role in fibrotic disorders affecting various different organs (myocardium, kidneys, liver, and lung), through the regulation of several processes including epithelial–mesenchymal transition (EMT), fibroblast activation, differentiation, and apoptosis, collagen synthesis and pro-fibrotic gene expression [7,8]. This huge involvement of miRNAs in pathological pathways makes them a promising therapeutic target in several conditions which still represent unmet medical needs [9,10].

In a preliminary study we identified, via computational analysis, a panel of candidate miRNAs that are possibly involved in BOS pathogenesis, and scored them according to the probability of them being involved in the condition [11]. We also documented a significant up-regulation of two of the most highly scoring miRNAs (miR-21-5p and miR-34a-5p) in a small number of lung samples of patients with BOS and OB in comparison with normal lung tissues, supporting their involvement in fibroblast activation [11].

The present study aims to extend the study initiated by Di Carlo et al. with the in vivo validation of other miRNAs that are potentially involved in BOS, and to further investigate the role of miR-21-5p and its pathway in BOS. The final objective of this exploratory study is to identify miRNAs which could serve as early biomarkers and/or therapeutic targets in BOS.

## 2. Materials and Methods

### 2.1. Tissue Samples

Archival formalin-fixed, paraffin-embedded (FFPE) samples of lung grafts from 12 patients who developed clinically diagnosed and histologically proven OB and/or RAS were collected from the University of Padua, the IRCCS Foundation Policlinico San Matteo, the University of Pavia, the Department of Pulmonary Medicine, the Translational Medicine Federation of Strasbourg and the University Hospital of Strasbourg. The study was approved by the Research Ethics Committee of the University of Padua (Protocol No. 0004959 27 January 2011) and by the Pavia Area Ethics Committee of the IRCCS Foundation Policlinico San Matteo (Protocol No. 20140003328: 28 July 2014). Samples provided by Strasbourg University Hospital were treated according to the French legislation on explanted material. Patient written informed consent was obtained before explanting. All lung tissues underwent 24 h formalin fixation before sampling and routine paraffin embedding; archival time ranged between 1 and 15 years. Sections stained with hematoxylin and eosin and Movat pentachrome were reviewed by an experienced histopathologist to classify the histopathological features of CLAD [12,13,14]. Archival formalin-fixed, paraffin-embedded normal lung tissue samples from organ donors and lung resections performed for pT1 cancer were used as controls (n = 5). Finally, human fibroblast commercial cell line (1187) was used as a healthy control to normalize the expression of miRNAs.

### 2.2. miRNAs Selection

hsa-miR-21-5p, hsa-miR-34a-5p, hsa-miR-15a-5p, hsa-miR-145-5p and hsa-miR-146b-5p were selected from the ranked panel of candidates defined by Di Carlo et al., 2016 [11]. All these miRNAs showed the highest scores in the panel. We also decided to evaluate hsa-let-7d-5p because it was reported as being significantly downregulated in fibrotic lung disorders, both in an animal model of bleomycin-induced lung fibrosis and in human lungs with regular interstitial pneumonia [8,15].

### 2.3. In Situ Hybridization (ISH)

miRNA in situ hybridization (ISH) was performed on unstained FFPE sections according to previously reported protocols [11], with double DIG-labeled miRCURY LNA^®^microRNA Detection Probes (Exiqon Inc., Vedbaek, Denmark), hybridized at a concentration of 100 nM at 37 °C for 20 h. Small nuclear RNA, RNU6, and a scramble probe were used, respectively, as positive and negative reaction controls. Stained slides were analyzed by a pathologist to characterize the cell type and localization of expression and to provide a semi-quantitative evaluation of the intensity of expression.

### 2.4. RNA Extraction and Reverse Trascriptase-Polymerase Chain Reaction

Total RNA was isolated from FFPE tissue samples using miRCURY RNA isolation FFPE (Exiqon Inc., Vedbaek, Denmark). For cell RNA isolation we used the miRNeasy Mini Kit (Qiagen). RNA concentration and purity were evaluated using a spectrophotometer (Nanodrop 2000, Thermo Scientific, Madison, WI, USA). Total RNA was reverse transcribed with the miRCURY LNA Universal cDNA Synthesis Kit II (Exiqon Inc., Vedbaek, Denmark), according to the manufacturer’s instructions.

### 2.5. miRNA Expression Analyses

qRT-PCR was performed with LNA probes (miRCURY LNA^TM^ miRNA PCR Assay, Qiagen, USA) and microRNA PCR ExiLENT SYBR Green master mix (Exiqon Inc., Vedbaek, Denmark) for all miRNAs. Expression levels of RNU6 were used as the normalization control. All reactions were performed on an LC480 Real-Time PCR system (Roche Diagnostics, Vienna, Austria) according to the manufacturer’s recommendations. Each experiment was performed in triplicate. The threshold cycle (Ct) was defined as the fraction cycle number at which fluorescence exceeded the given threshold. Relative quantifications were calculated with the comparative Ct method (2^−ΔCt^).

### 2.6. Computational Analysis of miR-21-5p Interactors

In light of the involvement of miR-21-5p in several pulmonary and extra-pulmonary fibrotic processes, we decided to analyze its interaction network to clarify the possible pathophysiological mechanisms at the base of BOS. DisGeNET v4.0 (https://disgenet.org, accesed on 17 February 2020), a discovery platform integrating gene–disease associations from several public data sources and the literature, was used to computationally select the set of target genes associated with BOS development (disease identification in DisGeNET- UMLS CUI: umls:C0006272). The CyTransfinder software [16] was used to infer direct interactions between miR-21-5p and the identified set of genes. Indirect interactions between miR-21-5p and selected genes were also analyzed using CyTransfinder; miR-21-5p targets were identified in 5 public miRNA target repositories including miRTarBase (http://miRTarBase.cuhk.edu.cn/ accesed on 21 February 2020), TargetScan (http://www.targetscan.org/vert_72/ accesed on 21 February 2020), PicTar 4 and 5 (https://pictar.mdc-berlin.de/ accesed on 24 February 2020), and miRanda (http://www.microrna.org/ accesed on 24 February 2020), and those that appeared in at least to 2 of these were investigated for their association with BOS-related genes listed in the DisGeNET database.

### 2.7. Immunohistochemistry

Immunohistochemical stains were performed on unstained FFPE sections with antibodies directed against STAT3 on an automated BenchmarkXT autostainer platform (Ventana, Tucson, AZ), using the ultraView Universal DAB revelation system (Ventana). Stained slides were analyzed by the study pathologist to register the cell type and site of expression and to give a semiquantitave evaluation of intensity.

### 2.8. Cell Culture and miR-21-5p Inhibition

Lung fibroblasts (LFs) were isolated from the bronchoalveolar lavage fluids (BAL)—performed for diagnostic purposes—of three patients with BOS and one lung transplanted patient without CLAD, following the procedure published by Cova et al., 2015 [17]. In brief, 6 × 10^6^ cells were seeded in high glucose Dulbecco’s modified Eagle medium (DMEM) with 10% fetal bovine serum (FBS), 100 U/mL penicillin/streptomycin (P/S) solution and 100 U/mL L-glutamine. Single foci of LFs formed between 7–28 days were isolated and cultivated. For the experiments, cells between the second and fifth passage after isolation were used. The study was approved by the ethics committee and each patient signed informed consent (prot.20200046007). A549 cell lines (ATCC^®^, Manassas, VA, USA) were cultivated in in the same medium. To induce the inhibition of miR-21-5p expression LFs and A549 were incubated for 4 h with 50 nM of anti-miR21 using INTERFERin^®^ (Polyplus transfection S.A. Illkirch, France) in FBS-free medium. Then, the cells were washed and incubated for 48 h, 72 h, 96 h with complete medium. Therefore, RNA was isolated from cells and miR-21 expression levels were evaluated as described above. To evaluate STAT3 gene expression, cDNA was retrotranscribed from 1 µg of total RNA using LunaScript RT SuperMix Kit (NEB). Relative levels of STAT3 mRNA were assessed using SYBR^®^ Green Luna^®^ Universal qPCR Master Mix (NEB) and normalized to the levels of glyceraldehyde-3-phosphate dehydrogenase (GAPDH) mRNA. Relative gene expression level quantification was compared with internal standards and analyzed using the 2^−ΔΔCt^ method.

### 2.9. Western Blot Analysis

LFs of BOS patiens teated with anti-miR21 for 48 h were collected for total protein extraction. Cells were washed with PBS, lysed with lysis buffer (50 mM Tris-HCl (pH 7.4), 150 mM NaCl, 10% glycerol, 1% NP-40, protease inhibitor cocktail (Sigma Aldrich, St. Louis, MI, USA) and phosphatase inhibitor (Roche), gently vortexed for 20 min at 4 °C and centrifuged for 15 min at 13,200 rpm at 4 °C. Supernatants were quantified by Pierce™ BCA Protein Assay Kit (Thermo Fisher Scientific, Waltham, MA, USA). Thirty micrograms of proteins from LFs extracts were loaded and separated in 8% SDS-PAGE. After electrophoresis, the gels were transferred to polyvinylidene difluoride membranes (Millipore, Burlington, MA, USA) and, therefore, blocked (5% no fat milk in 0.1% Tween 20 TBS) and incubated with the primary Ab (1:1000 in TBST + 2% BSA; overnight at 4 °C or 2 h at room temperature): anti-STAT3 monoclonal antibody [9D8] (MA1-13042, Invitrogen, Carlsbad, CA, USA) and anti-β-Actin (MAB1501R, Chemicon, Kitakami, Japan). After washing, the membranes were incubated with the appropriate horseradish-peroxidase conjugated secondary Ab (1:5000 in TBST + 2% BSA; 2 h at room temperature; anti-mouse A4416, Sigma, Burlington, MA, USA). The immunoreactivity was detected by ECL reagents (Amersham, London, UK), acquired with the ChemiDoc imaging system (Image Lab, Bio-Rad, Berkeley, CA, USA).

### 2.10. Statistical Analysis

All results are presented as mean ± standard deviations. Statistical significance of observed differences for continuous variables among different groups was calculated using parametric or non-parametric tests, according to data distribution. All tests were two-tailed. Differences at *p* < 0.05 were considered to be statistically significant.

## 3. Results

### 3.1. Patient Data

The clinical features of the studied patients are listed in Table 1. Of the patients studied, 83% were male with a mean age at first transplant of 35.25 ± 14.06. Patients studied underwent re-transplantation 65.75 ± 41.48 months after the first LTx and at the time of the second LTx were on triple immunosuppressant therapy. All patients developed CLAD after the first LTx and underwent re-transplantation. Histopathological revision of the explanted lungs documented that: (1) all samples showed partial or complete OB; (2) in four samples, there was prominent obliteration of medium and small-sized vessels (hyperplastic vasculopathy); (3) four samples presented the pattern of interstitial fibrosis associated with RAS; (4) in one case, there was also evidence of lymphocytic bronchiolitis. Notably, four samples showed both histological phenotypes of CLAD (OB and interstitial fibrosis).

### 3.2. In Situ Hybridization

**Figure 1:**
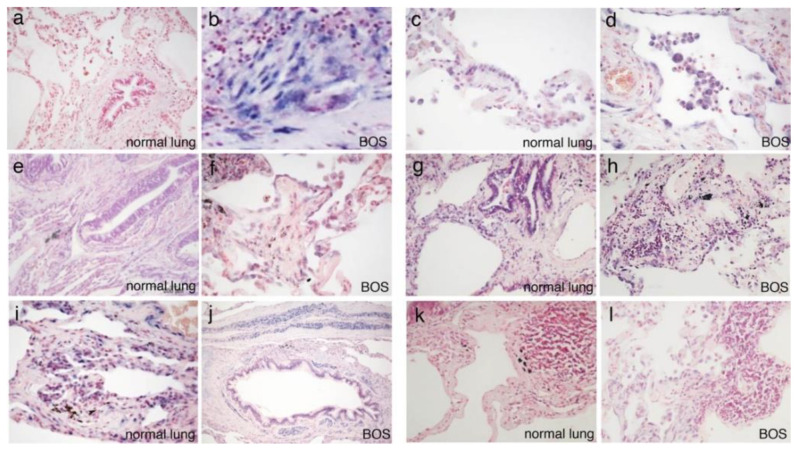
Tissue localization of miRNAs in normal lung (miR-21-5p (**a**); miR-146b-5p (**c**); miR-34a-5p (**e**); miR-15a-5p (**g**); miR-145-5p (**i**); let-7d-5p (**k**)) and BOS samples (miR-21-5p (**b**); miR-146b-5p (**d**); miR-34a-5p (**f**); miR-15a-5p (**h**); miR-145-5p (**j**); let-7d-5p (**l**)) by ISH.

ISH tests confirmed the results presented by Di Carlo et al., 2016 [11], on the expression of miR-21-5p (Figure 1a and Table 2) and miR-34a-5p (Figure 1b and Table 2) in transplanted lungs. miR-21-5p showed de novo intense expression in the fibroblasts of obliterative lesions and interstitial fibrosis; miR-34a-5p was diffusely expressed in several cell types of normal lung tissues as well as in OB and interstitial fibrosis [11]. The other considered miRNAs were similarly expressed in fibroblasts and myofibroblasts of bronchiolar and interstitial fibrotic lesions in transplanted lung sections and in normal lungs (Table 2). Specifically, all of them were expressed in bronchial and reactive alveolar epithelia, and in endothelia. miR-34a-5p, miR-15a-5p and miR-146b-5p were also expressed in macrophages, and let-7d-5p in plasma cells; finally, miR-145-5p, and to a lesser extent miR-15a-5p, stained airway and vascular smooth muscle cells.

### 3.3. miRNA Expression Levels

**Figure 2:**
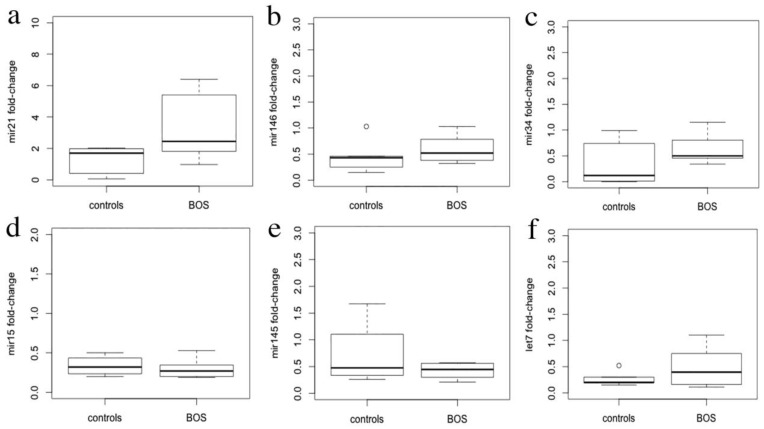
Quantitative analysis of miRNAs expression. qRT-PCR miRNAs expression levels in BOS samples, mir-21 (**a**), mir-146 (**b**), mir34(**c**), mir15 (**d**), mir145 (**e**), let7 (**f**), represented as fold changes relative to RNU6.

The expression levels of miR-21-5p, miR-34a-5p, miR-15a-5p, miR-145-5p, miR-146b-5p and let-7d-5p determined by qRT-PCR in total RNA isolated from FFPE samples are represented in Figure 2. Only miR-21-5p levels were significantly different in transplanted lung samples, showing increased expression as compared with normal lung tissue (*p* = 0.02).

### 3.4. Computational Identification of miR-21-5p Targets and Immunohistochemistry

**Figure 3:**
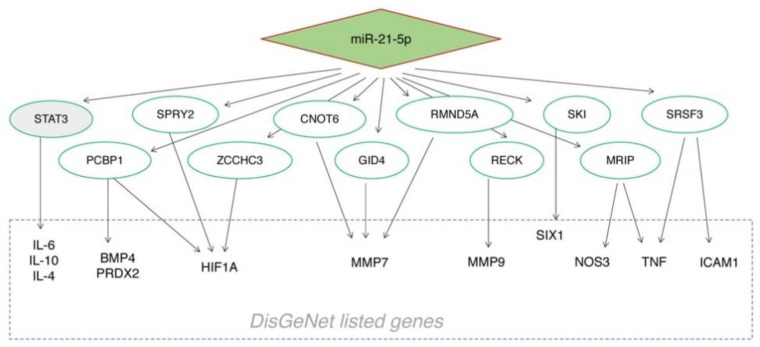
miR-21-5p regulatory network. Interactions between miR-21-5p and genes that appeared to be associated with BOS in at least 3 different databases (Mirtarbase, Targetscan, Miranda, Pictar4, and Oictar5). miR-21-5p-related genes (green boxes) encoding for proteins that interact with BOS-related genes listed in DisGeNet database (light blue boxes).

**Figure 4:**
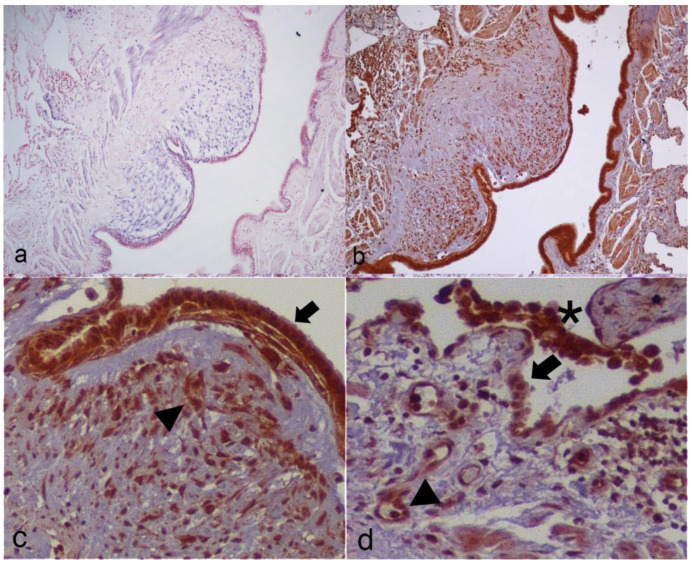
Tissue localization of miR-21-5p and STAT3 in OB lesions. Photomicrographs representative of the co-localization of miR-21-5p (**a**) and STAT3 (**b**) in fibroblast of OB lesions and in the adjacent tissue. High magnification pictures show details of STAT3 expression in (**c**) bronchial epithelium (arrow) and OB fibroblast (arrowhead), and in (**d**) alveolar pneumocytes (arrow), endothelia (arrowhead), and macrophages (asterisk).

In the DisGeNET database, 63 genes were found to be annotated as associated with BOS (http://www.disgenet.org/browser/0/1/0/C0006272/0/25/63/_a/_b./-score/ accessed on 17 February 2020). By CyTransfinder, no direct interaction was found between miR-21-5p and DisGeNET listed genes. Therefore, we decided to first explore the interactors of miR-21-5p using five public miRNA databases, and selected those that appeared in at least three, four and five databases (Table S1), to further check their association with BOS-related genes listed in DisGeNET database [18]. STAT3 and PCBP1 showed more interactors in the DisGeNet database (Figure 3). Between these, only STAT3 showed a documented involvement in several fibrotic processes [19], with a role in bleomycin-induced lung fibrosis [20] and idiopathic pulmonary fibrosis (IPF) [21]. The STAT3 expression profile was tested with immunohistochemistry in transplanted lungs, showing positive expression of STAT3 in fibroblastic obliterative lesions and de novo expression of STAT3 in endothelia and smooth muscle cells as compared to normal lungs. STAT3 was also expressed in the bronchial epithelia, alveolar epithelia and alveolar macrophages (Table 3, Figure 4).

### 3.5. miR-21-5p Inhibition in Cell Cultures

**Figure 5:**
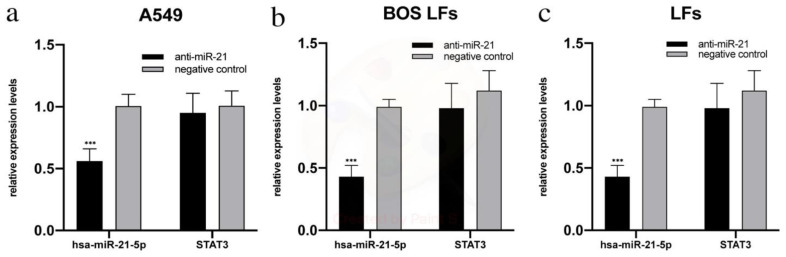
qRT-PCR analysis of miR-21-5p/STAT3 mRNA expression levels: (**a**) in A549, mean ± standard deviations of replicates; (**b**) in Lung fibroblasts (LFs) derived from BOS patients, mean ± standard deviations of all cell lines; (**c**) in LFs derived from patients without chronic lung allograft dysfunction (CLAD), mean ± standard deviations of replicates. *** *p* < 0.001.

To test the positive feedback loop between miR-21-5p and STAT3 documented in other diseases, such as cancer [20,21,22], we inhibited the expression of miR-21-5p in A549 cells and LFs derived from the BAL of patients with and without CLAD. RNA isolated from these cells was used to evaluate the expression of miR-21-5p and STAT3 mRNA. Treatment with anti-mir-21 in LFs induces a reduction in miR-21-5p levels after 48h of treatment, as shown in Figure 5 (*p* < 0.001). After 72 h of transfection, the levels returned to values that were not significantly different to those of the untreated control and remain at such levels even after 96 h (data not shown). Efficient reduction in miR-21-5p levels is evident even 48 h after treatment in A549. Despite the down-regulation of miR-21-5p with a significant reduction in miR-21-5p levels after 48 h of anti-miR treatment, STAT3 mRNA levels were not significantly affected in all cell lines tested (Figure 5). STAT3 mRNA levels do not change even at later times after treatment.

**Figure 6:**
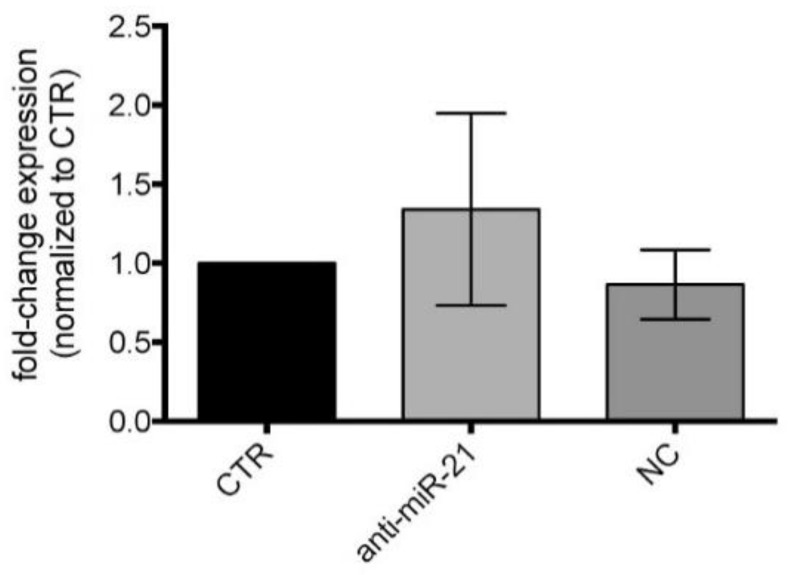
Western blot of STAT3 protein expression levels in LFs treated with anti-miR-21. Quantification of immunoblots using anti-STAT3 of LFs from BAL of CLAD patients treated with anti-miR-21 after 48h. Control cells (CTR); negative control treated cells (NC).

We also validated the protein level of STAT3 in LFs derived from the BAL of patients with CLAD, showing that, after 48h of anti-miR treatment, STAT3 protein levels were not significantly affected in the cell lines tested compared to control cells (CTR) and negative control (NC) treated cells (Figure 6).

## 4. Discussion

In this study a panel of candidate miRNAs possibly involved in BOS pathogenesis was tested by qRT-PCR and ISH in the lungs of patients with CLAD. Quantitative analysis showed that only the level of miR-21-5p was significantly higher in BOS lungs than in the control, while in situ characterization provided further information on the distribution of the investigated miRNAs in affected lungs. miR-21-5p showed high disease- and lesion-specific expression in OB and RAS fibroblasts. This observation supports the correlation between de novo miR-21-5p overexpression and the fibro-obliterative process underlying CLAD, along with other fibrosing disorders, and confirms our preliminary results [11].

miR-21-5p dysregulation has been documented in different pulmonary fibrotic processes [20,23,24]. Its levels are increased in animal models of bleomycin-induced pulmonary fibrosis [20], in fibroblast foci [24], and also in the serum of patients with idiopathic pulmonary fibrosis [25,26]. Several studies have tried to understand the exact role of miR-21-5p in lung fibrosis. In this context, one of the suggested possible hypotheses is that this miRNA seems to be involved in the tumor growth factor (TGF)-β1 signaling pathway which regulates EMT, which is relevant in the process [23]. As far as BOS is concerned, miR-21-5p serum levels are increased in lung transplant patients before the onset of BOS [27], however, it is not clear whether miR-21-5p can be considered a potential therapeutic target for BOS, or it could at least be exploited as a prognostic marker for BOS onset in lung transplanted patients.

In this study, using DisGeNET together with CyTransfinder software, we observed that miR-21-5p does not directly interact with any of the BOS-related genes listed in DisGeNET. For this reason, we decided to consult different miRNA databases in order to find which proteins were more likely related to miR-21-5p. Subsequently, we investigated their association with the BOS-related genes listed in DisGeNET. After computational analysis, we identified eleven miR-21-5p-related genes that encode proteins that interact with various BOS-related genes. Some of these reported targets are cancer-related and are associated with carcinogenesis (such as SPRY2, SKY and SRSF3), cancer prognosis (such as CNOT6, RECK and GID4) or cancer cell plasticity (such as RMND5A) [28,29,30,31]. Interestingly, between these targets only STAT3 and PCBP1 showed more interactors. STAT3 regulates fundamental cellular processes, including inflammation, cell growth, proliferation, differentiation, migration, and apoptosis. Previous studies have demonstrated that STAT3 exerts anti-fibrotic effects in many fibrosis diseases, such as systemic sclerosis (SSc), skin fibrosis, and renal fibrosis [32,33,34]. In addition, STAT3 activation is detected in lung biopsies from patients with idiopathic pulmonary fibrosis and bleomycin (BLM)-induced murine fibrotic lungs [35,36]. Significant activation of STAT3 has also been demonstrated in both cultured lung fibroblasts upon TGF-β1 stimulation and silica-induced murine fibrotic lungs [37]. Nevertheless, its involvement in BOS has not previously been explored. In reverse, PCBP1 is involved in several cellular processes that, in particular, are related to iron metabolism and it is largely studied in cancer [38,39]. However, to the best of our knowledge, no involvement of PCPB1 in CLAD or fibrotic processes has been proven. The STAT3 expression profile in transplanted lungs showed positive expression of STAT3 in fibroblastic obliterative lesions and de novo expression of STAT3 in endothelia and smooth muscle cells as compared to normal lungs. These data appear to confirm the specific expression of this protein in the lungs of transplanted subjects and its absence in normal lungs, confirming the hypothesis of its involvement in BOS disease. Furthermore, by comparing in situ STAT3 expression and ISH analyses, we observed that STAT3 and miR-21-5p expression patterns overlap in OB lesions in transplanted lungs.

miR-21-5p expression is regulated by STAT3 through the presence of two phylogenetically conserved STAT3 binding sites in the miR-21-5 regulatory region [40]. At the same time, miR-21-5p can indirectly induce STAT3 by targeting PIAS3, a STAT3 inhibitor, forming a positive feedback loop [41,42,43]. To further study the relationship between STAT3 and miR-21-5p, and more specifically, to confirm the presence of the STAT3/miR-21-5p positive feedback loop mediated by the down-regulation of STAT3-inhibitor by miR-21-5p in BOS, we evaluated STAT3 mRNA levels in cultured LFs derived from BAL and A549 cells after miR-21-5p inhibition. In our study we have demonstrated that miR-21-5p inhibition treatment induces a reduction in miR-21-5p levels after 48 h of treatment with anti-miR-21. However, this does not lead to the alteration of both STAT3 mRNA and protein expression levels, even though STAT3 is described in many databases as a target of miR-21-5p. This could be due to the fact that STAT3 expression regulation is determined by a complex pathway, in which miR-21-5p is not the only actor. This might also suggest that miR-21-5p expression is regulated by STAT3 at the transcriptional level. Indeed, it has been previously shown that STAT3 activation is required for miR-21-5p expression [22], as STAT3 binds multiple binding sites in the miR-21-5p promoter and is required for transcription induction [42]. Moreover, in human brochial epithelial cells exposed to arsenite, it was observed that IL-6 mediates the increase in miR-21-5p levels, inducing STAT3 activation [43]. Further studies will be needed to determine the precise role of STAT3/miR-21-5p interaction and the pathway underlying this in CLAD. It has to be acknowledged, as a limitation of our study, that we used the A549 type II pneumocyte cell line of human lung adenocarcinoma, and not fibroblasts. Despite this consideration, the A549 cell line allowed us to verify the miR-21-5p inhibition efficiency following transfection, without altering the levels of STAT3 mRNA and, thus, confirming our data in LFs derived from BAL.

Several published studies have suggested that the other miRNAs selected based on our previous computational analysis are implicated in fibrosis in different models and settings, including CLAD [4,7,44]. While quantitative analyses have not shown a significant increase in expression levels in transplanted lung samples with respect to normal lung tissues, our combined approach documented their expression at the cellular level in fibroblasts and myofibroblasts of bronchiolar and interstitial fibrotic lesions, and of vascular obliterative proliferations. These results show that, from a methodological point of view, in situ hybridization and qRT-PCR are complementary approaches and can be usefully employed to assess miRNA dysregulation. The in situ analysis, although qualitative, has the advantage of precisely assessing the site (cell type/tissue component) of miRNA expression, and integrates the quantitative data obtained by qRT-PCR within the pathological background of the diseases. This observation is particularly relevant in topographically heterogeneous diseases such as OB, the lesions of which may involve a relatively small proportion of lung tissue. In these conditions, a purely quantitative approach could miss significant expression changes that occur in limited, although highly disease-specific, tissue components. Nonetheless, the complex pathway regulation of other miRNAs requires dedicated investigation.

## 5. Conclusions

Our results indicate that, among the tested miRNAs, the pattern of expression of miR-21-5p is strongly suggestive of this miRNA playing a role as a pro-fibrotic effector in CLAD fibro-obliterative processes. Furthermore, this process is possibly mediated by activation of the STAT3 pathway. Our research also indicates the presence of a complex regulatory pathway involving miR-21-5p, which does not seem to be solely responsible for regulating the STAT3 pathway. Moreover, the present research underlines the utility of a combined methodological approach for the study of miRNA dysregulation in tissues, and the advantages of direct in situ localization of dysregulated miRNAs at the cellular/subcellular level.

## Figures and Tables

**Table 1:** Clinical data of studied patients.

Case	Sex	Age at First LTx	Original Diagnosis	Months to Retransplant	Pathological Pattern of CLAD ^g^
Str-1	M	66	NSIP ^a^ (Sjogren Syndrome)	28	OB ^h^, RAS ^i^, HV ^l^
Str-2	M	51	UIP ^b^	27	OB, HV
Str-3	M	28	CF ^c^	32	OB, HV
Str-4	F	27	CF	1	OB
Str-5	M	33	BOS ^d^	115	OB
Str-6	M	20	CF	43	OB, RAS
Str-7	M	18	CF	41	OB, RAS
Pav-1	M	50	UIP	90	OB, RAS, HV
Pav-2	F	36	CF	100	OB, LB ^m^
Pad-1	M	37	CLL ^e^	82	OB, LB
Pad-2	M	31	CML ^f^	104	OB
Pad-3	M	26	CF	126	OB

^a^: non-specific interstitial pneumonia; ^b^: usual interstitial pneumonia; ^c^: cystic fibrosis; d: bronchiolitis obliterans syndrome; ^e^: chronic lymphocytic leukemia; ^f^: chronic myeloid leukemia; ^g^: chronic lung allograft dysfunction; ^h^: bronchiolitis obliterans; ^i^: restrictive allograft syndrome; ^l^: hyperplastic vasculopathy; ^m^: lymphocytic bronchiolitis.

**Table 2:** Topographic and semi quantitative evaluation of miRNAs with in situ hybridization (ISH).

	Type II Pneumocyte		Bronchial Epithelia		Endothelia		Bronchial Smooth Muscle		Vascular Smooth Muscle		Myofibroblasts	Inflammatory Cells	
	NL	TL	NL	TL	NL	TL	NL	TL	NL	TL	TL	NL	TL
miR-21-5p	-	+	-	+	-	+	-	++	-	+	+++	M	M
miR-34a-5p	+	+	+	+	-	+	-	-	-	-	+	M	PC
miR-15a-5p	+++	+++	+++	+++	++	++	++	++	++	++	+++	L, M	L, M
miR-145-5p	+	+	++	++	+	+	+++	+++	+++	+++	+	-	-
miR-146b-5p	+	+	+	-	-	-	-	-	-	-	+	M	PC
Let-7d-5p	++	+++	+++	+++	++	++	-	-	-	+	++	PC	PC

NL = normal lungs; TL = transplanted lungs; M = macrophages; L = lymphocytes; PC = plasma cells.

**Table 3:** Topographic and semi quantitative evaluation of STAT3.

	Type II Pneumocytes		Bronchial Epithelia		Endothelia		Bronchial Smooth Muscle		Vascular Smooth Muscle		Myofibro	Inflammatory Cells	
	NL	TL	NL	TL	NL	TL	NL	TL	NL	TL	TL	NL	TL
STAT3	+	++	++	++	-	+	-	++	-	+	+++	M, PC	M, PC

NL = normal lungs; TL = transplanted lungs; M = macrophages; PC = plasma cells.

## Data Availability

The data presented in this study are available on request from the corresponding author.

## Supplementary Materials

The following are available online at https://www.mdpi.com/2073-4409/10/3/688/s1, Table S1: miR-21-5p interactors founded repeated in considered data bases.
