# Supplementary material for: miRNAs Potentially Involved in Post Lung Transplant-Obliterative Bronchiolitis: The Role of miR-21-5p
Source: Cells. 2021 Mar 20;10(3):688. doi: 10.3390/cells10030688 (PMC8003603; doi:10.3390/cells10030688)
Supplement: Supplementary file 1 [file cells-10-00688-s001.pdf]

Table S1: miR-21a-5p interactors founded repeated in considered data bases

**At least 3**				**At least 4**			**At least 5**
ASPN	FASLG	PAN3	SCRN1	ADNP	MBNL1	SATB1	SPRY2
BAHD1	GATAD2B	PBRM1	SMARCD1	AP1AR	MPRIP	SKI	JAG1
BCL11A	HNRNPK	PCSK6	SOX2	ARHGAP24	NFIB	SMAD7	PDCD4
BCL2	HRB1	PDZD2	SPRY1	BTG2	NTF3	STAG2	SOX5
BMPR2	KBTBD6	PER2	SRSF3	CDC25A	PCBP1	TAGAP	
BNC2	LEMD3	POM121	STAT3	CHD7	PELI1	TGFBI	
BOLL	LRRC57	PPARA	STK40	CNOT6	PITX2	TIMP3	
CASKIN1	MATN2	PPP3CA	TGFBR2	CREBRF	PLAG1	WWP1	
CCL1	MRPL9	PURB	TIAM1	ELF2	PLEKHA1	XKR6	
CNTFR	MTAP	RAB11A	TOPORS	FBOX11	RASGRP1	YAP1	
CPEB3	MTMR12	RASA1	TRAPPC8	GID4	RHOB		
CREBL2	NBEA	RECK	TRPM7	KRIT1	RMND5A		
EHD1	OSR1	RNF111	UBR3				
EPHA4	PAG1	RP2	ZADH2				
			ZCCHC3				
			ZNF367				
